# Probiotics as Dietary Supplements for Eradication of *Helicobacter pylori* Infection in Children: A Role Beyond Infection

**DOI:** 10.3390/children3040027

**Published:** 2016-11-10

**Authors:** Sherif T. S. Hassan, Miroslava Šudomová

**Affiliations:** 1 Department of Natural Drugs, Faculty of Pharmacy, University of Veterinary and Pharmaceutical Sciences Brno, Palackého tř. 1946/1, 612 42 Brno, Czech Republic; 2 Museum of the Brno Region, Museum of Literature in Moravia, Porta Coeli 1001, 66602 Předklášteří, Czech Republic; sudomova@post.cz

**Keywords:** probiotics, *Helicobacter pylori* infection, eradication treatment, dietary supplements, children

## Abstract

For decades, treatment of infectious diseases has been a strong focus of interest, for both researchers and healthcare providers. Chronic infection with *Helicobacter pylori* (*H. pylori*) has been reported to be associated with several diseases, such as ulcer disease, gastric adenocarcinoma and mucosa-associated lymphoid tissue (MALT) lymphoma. Infection with *H. pylori* is generally acquired during childhood and can persist indefinitely, if not treated systematically. Unfortunately, although several strategies have shown high efficacy results, treatment of the *H. pylori* infection fails in about 25%–30% of infected children. One main reason for this is due to the extensive use of antibiotics, which has created antibiotic resistance, associated with other adverse effects as well. Therefore, it is crucial to find alternative strategies to combat this resistance, and increase treatment efficacy results. Probiotics, which are live microorganisms that are orally administrated, have been found to be a useful regimen in the treatment of the *H. pylori* infection in children. Their use as a dietary supplement alone, or in combination with antibiotics, resulted in reduced side effects and higher efficacy rates of the *H. pylori* infection in children. Some probiotics can be considered an adjunctive treatment, especially when eradication of the *H. pylori* infection fails during initial treatment, and to help reduce adverse effects. However, the evidence of the beneficial role of probiotics is limited due to the small number of clinical trials that have been conducted and heterogeneity across studies in strains and dosage. Additionally, no investigations have been carried out in asymptomatic children. Therefore, large well-conducted studies are needed to evaluate the efficacy and safety of probiotics as an adjuvant therapy of the *H. pylori* infection.

## 1. Introduction

*Helicobacter pylori* (*H. pylori*) is a gram-negative bacterium that colonizes the human stomach. This pathogen is known to be a causative agent in peptic ulcer, gastric adenocarcinoma, and extranodal marginal cell lymphoma of the stomach. The infection is acquired mainly in childhood and is associated with diverse gastrointestinal symptomatology, including recurrent abdominal pain, chronic hemorrhagic gastritis, and follicular gastritis [1,2,3,4]. *H. pylori* is frequently associated with dyspepsia, one of the most common upper gastrointestinal complaints. Since chronic follicular gastritis in childhood can increase the risk for developing gastric neoplasia in adult life [5,6], it is very important to focus attention on this infection, especially in childhood. In most cases, physicians recommend the use of antibiotics as a first line treatment. Although antibiotics can often effectively treat the infection, this therapy has several important limitations, such as the problem of drug-resistant strains, adverse side effects, and high costs [7,8,9].

## 2. Alternative Therapy

The use of probiotics as potential anti-infective microorganisms has now been suggested as an alternative therapy for the *H. pylori* infection, which provides several advantages, such as reduced side effects, less resistance, and various mechanisms of action [10]. Probiotics are beneficial, live microorganisms and can be used either as single species or as a multispecies preparation. The beneficial effects of probiotics appear to be strain-specific, as well as in a dose dependent manner. Probiotic monotherapy has been shown to effectively decrease *H. pylori* density (expired ^13^CO_2_) by 2%–64%. Moreover, probiotic monotherapy has also been shown to eradicate *H. pylori* in up to 32.5% of infected cases, although subsequent recrudescence is likely [11,12,13,14].

The most frequently used strains in the majority of *in vivo* or human studies were *Lactobacillus johnsonii La1* and *Lactobacillus rhamnosus GG* (either in a fermented milk preparation containing live bacteria, or as a cell-free culture supernatant), followed by other commonly used probiotics, such as *Lactobacillus acidophilus, Lactobacillus gasseri OLL2716*, *Lactobacillus casei*, *Lactobacillus reuteri*, *Lactobacillus brevis*, and *Bifidobacterium breve*, *Bifidobacterium animalis*, *Bifidobacterium lactis*, *Propionibacterium freudenreichii*, along with the probiotic yeast *Saccharomyces boulardii* [15,16,17]. Various tests can be used to determine the effect of probiotics on the *H. pylori* infection, such as urea breath test, rapid urease tests, stool antigen test, and histological examination of gastric biopsies and serological assays [17,18].

Several clinical trials have been conducted on probiotics, in order to determine their use as a complement during *H. pylori* treatment in children (Table 1). For instance, a randomized double-blind placebo-controlled study was performed to evaluate the efficacy of triple therapy (amoxicillin, clarithromycin, omeprazole), supplemented with a fermented milk product containing a *Lactobacillus casei* (*L. casei*) DN-114 001 strain, on *H. pylori* treatment in 86 dyspeptic children. The results demonstrated that supplementation of fermented milk containing live probiotic *L. casei* DN-114 001 (1 × 10^10^ CFU/day for 14 days) with triple therapy, confers an enhanced therapeutic benefit on *H. pylori* eradication in children [19]. In another study, 40 dyspeptic children were involved in a randomized double-blind placebo-controlled trial. It was found that the use of *Lactobacillus reuteri* ATCC 55730 (capsule, 1 × 10^8^ CFU/day for 20 days) in combination with 10-day sequential therapy (omeprazole + amoxicillin for the first 5 days, and omeprazole + clarithromycin + tinidazole for the following 5 days), led to significant decreases of antibiotic-associated side effects [20]. In another randomized clinical trial (RCT), 65 children were treated for one week with amoxicillin, clarithromycin plus omeprazole, and probiotic food consisting of 250 mL of a commercial yogurt containing *Bifidobacterium animalis* (250 mL yogurt, 10^7^ CFU/mL). The results revealed that the use of probiotic food, in combination with antibiotics, effectively eradicated the *H. pylori* infection in children [21].

Additionally, four meta-analyses of RCT’s have been performed to determine the efficacy of probiotics in *H. pylori* eradication therapy in children (Table 2). The results showed that the supplementation of probiotic strains (e.g., *Saccharomyces boulardii*, *Lactobacillus* or *Bifidobacterium* strains) with triple therapy (amoxicillin, clarithromycin, omeprazole), effectively increased the eradication rate of *H. pylori,* in comparison with a monotherapy of two antibiotics plus a proton pump inhibitor. Moreover, the addition of probiotics reduced side effects from the antibiotic therapies with significant heterogeneity, particularly diarrhea [22,23].

## 3. Concluding Remarks

Products containing probiotic strains may serve as a useful tool to increase eradication of an *H. pylori* infection in children, when properly used as a complement to the first- or second-line eradication therapy. Furthermore, probiotic combinations can reduce adverse side effects induced by antibiotics, although continuing studies comprised of a larger number of patients, are necessary to further evaluate the efficacy of probiotics as a supplement of antibiotic therapy, in the treatment of the *H. pylori* infection [24,25]. Here, the cooperation of healthcare providers, researchers, patients (children) and their parents is highly appreciated to perform such efforts.

## Figures and Tables

**Table 1:** Clinical trials using probiotics as dietary supplements during *Helicobacter pylori* (*H. pylori*) infection therapy.

Patient Group	Study Design	Eradication Treatment	Probiotic Strain	Type of Intervention	Treatment Outcome	Ref.
Dyspeptic children (*n* = 86)	DBPC, R	CA+ omeprazole	*Lactobacillus casei* DN-114 001	Fermented milk, 1 × 10^10^ CFU/day, 2 weeks	Increase of eradication rate and decrease of adverse effects, particularly of diarrhea by 53%	[19]
Dyspeptic children (*n* = 40)	DBPC, R	A+ omeprazole 5 days following CT+ omeprazole 5 day	*Lactobacillus reuteri* ATCC 55730	Capsule, 1 × 10^8^ CFU/day, 20 days	Increase of eradication rate and decrease of adverse effects, particularly of diarrhea by 65%	[20]
Children (n = 65)	R	CA+ omeprazole	*Bifidobacterium animalis*,	250 mL yoghurt, 10^7^ CFU/mL	Increase of eradication rate	[21]

A: amoxicillin; C: clarithromycin; CFU: colony forming units; DBPC: double-blind placebo controlled; R: randomized; T: tinidazole.

**Table 2:** Meta-analyses of probiotics supplementation in children.

Patient Group	Study Design	Eradication Treatment	Probiotic Strain	Treatment Outcome	Ref.
915 children	4 RCTs	Triple therapy	*Saccharomyces boulardii*	Increase of eradication rate (RR 1.13, 95% CI 1.05–1.21)	[22]
1305 children	5 RCTs	Triple therapy	*Saccharomyces boulardii*	Reduce the risk of overall *H. pylori*-therapy related adverse effects (RR 0.46, 95% CI 0.3–0.7)	[22]
1215 children	4 RCTs	Triple therapy	*Saccharomyces boulardii*	Reduce antibiotic- associated diarrhea (RR 0.47, 95% CI 0.32–0.69)	[22]
508 children	5 RCTs	Triple therapy	*Lactobacillus* or *Bifidobacterium* strains	Increase of eradication rate and reduced side effects of antibiotic therapies (OR = 0.32; 95% CI 0.13–0.79) with significant heterogeneity, particularly diarrhea (OR = 0.16; 95% CI 0.06–0.45)	[23]

RCT: randomized clinical trial; RR: relative risk; CI: confidence interval; OR: odds ratio.
